# Special Issue “EUV and X-ray Wavefront Sensing”

**DOI:** 10.3390/s22103940

**Published:** 2022-05-23

**Authors:** Mourad Idir, Daniele Cocco, Lei Huang

**Affiliations:** 1 Optical Metrology Group, Photon Science Division, Energy Sciences Directorate, Brookhaven National Laboratory-NSLS II, Upton, NY 11973-5000, USA; lhuang@bnl.gov; 2 Advanced Light Source, Lawrence Berkeley National Laboratory, Berkeley, CA 94720, USA; dcocco@lbl.gov

## 1. Introduction

X-ray optics are extensively used in synchrotron radiation and free-electron laser facilities, as well as in table-top laboratory sources. They are used to collimate, focus or, in general, to manipulate the X-ray beams, ideally at the diffraction limit and with high efficiency. The successful exploitation of the extreme-quality X-ray beams depends, to a significant extent, on the imperfections and misalignment of the optics employed. With the advent of fourth-generation storage rings and diffraction-limited sources, at-wavelength metrology is becoming more and more important to take full advantage of the extraordinary new characteristics of these high-brilliance sources. Their use becomes even more critical with the increasing use of active optics, the necessity to monitor and limit the thermal effects on X-ray optical elements, the determination of the X-ray beam wavefront itself, and the desire to achieve diffraction-limited and wavefront-preserving X-ray beams. The accuracy of ex-situ optical metrology has achieved continuous improvement in the last two decades, reaching what is probably a state-of-the-art level. The same may not be true for in-situ metrology. This limits the wavefront control on the beamlines, often affected by environmental and systematic alignment factors, as well as inadequate in-situ feedback.

This Special Issue (SI), entitled “*EUV and X-ray Wavefront Sensing*” is focused on original research performed by world-recognized scientists in the field and is focused on the current state-of-the-art in-situ wavefront sensing for EUV and X-ray. We believe that this SI will provide the reader with an overview of the present status and an outlook on future development in this field. The contributions to this SI resulted in a collection of eight published manuscripts, reporting on the advances in research related to different wavefront-sensing technologies, including their design and implementation.

A short overview of the collection of papers accepted for publication in this SI is presented in Section 2.

## 2. Contributed Papers

Following a rigorous review process, eight papers were selected. They cover a wide spectrum of research topics in the broader areas of this Special Issue. The manuscripts accepted for publication mirror the relevance of the topic for the research community, and the vast field of research that still exists to be explored to enhance EUV and X-ray Wavefront Sensing.

The SI starts with an article [1] covering the general aspects of EUV and Hard X-ray Hartmann Wavefront Sensors, describing some specific applications. With more than 15 years of experience, the authors guide the readers in understanding the potentiality of the Hartmann technique and how its proper calibration and application can help in optimizing the performance of a beamline.

The SI continues with the second contribution [2], describing a novel way for creating a “pseudo non-invasive” wavefront technique. It is based on reflecting Hartmann masks, intermittingly inserted into the x-ray beam, providing one-dimensional information on the X-ray wavefront.

In the third [3] and fourth [4] contributions to this SI, the authors present studies of simulation methods. One of the papers presented a way to simulate the images formed by Fresnel Zone Plates (FZP) with different parameters. Imaging performances of FZP are numerically investigated. The second paper shows interesting simulations for the performance of the Hartmann sensor, for different degrees of coherence of the source.

The authors from [5] presented a study on an in-situ speckle-scanning-based technique for the measurement of the X-ray crystal diffraction wavefront and the characterization of the slope error of channel-cut crystals, with different surface characteristics. This method elucidates possibilities to take new high-resolution X-ray crystal diffraction wavefront measurement and provide feedback to crystal manufacturers to improve channel-cut fabrication.

The careful control of each source of error in the measurement of the distorted wavefront, associated with highly accurate differential coating deposition, to optimize the shape of the optics, permits the realization of a diffraction-limited spot in the hard X-ray. The entire process is described in the sixth contribution [6].

In the seventh contribution [7], the wavefront is analyzed to perform otherwise very challenging, if not impossible, metrology on capillary optics. The Special Issue closes with an article dedicated to small-spot optimization, in the presence of a relatively large numerical aperture [8]. The work is performed on a Free-Electron Laser and is aimed to the optimization of the focal spot for microscopy application.

The energy range spectrum covered by these eight contributions spans from the visible to the hard X-rays, showing the versatility of those techniques.

## 3. Outlook and Prospects

The set of papers published in this SI is just a small representation of the current research interest regarding EUV and X-ray Wavefront Sensing. As this field evolves, improving its range of detection, resolution and accuracy, new applications and higher accuracy detection levels will be achieved, and this will widen the application of these technologies to an even greater extent. When coupled with AI algorithms, other emerging fields of application can be sought, and a vast area of research will be pursued. The pursuit for truly non-invasive wavefront analysis systems, coupled with the most recent development of adaptive optics, may revolutionize the X-ray beamlines as we know them. In fact, this may open the door to a real-time closed loop feedback system, not only optimizing the beamline performance but maintaining it for a long time period during long experiments. 
